# Minimal Absent Words in Four Human Genome Assemblies

**DOI:** 10.1371/journal.pone.0029344

**Published:** 2011-12-29

**Authors:** Sara P. Garcia, Armando J. Pinho

**Affiliations:** 1 Signal Processing Laboratory, Institute of Electronics and Telematics Engineering of Aveiro, University of Aveiro, Aveiro, Portugal; 2 Department of Electronics, Telecommunications and Informatics, University of Aveiro, Aveiro, Portugal; Auburn University, United States of America

## Abstract

Minimal absent words have been computed in genomes of organisms from all domains of life. Here, we aim to contribute to the catalogue of human genomic variation by investigating the variation in number and content of minimal absent words within a species, using four human genome assemblies. We compare the reference human genome GRCh37 assembly, the HuRef assembly of the genome of Craig Venter, the NA12878 assembly from cell line GM12878, and the YH assembly of the genome of a Han Chinese individual. We find the variation in number and content of minimal absent words between assemblies more significant for large and very large minimal absent words, where the biases of sequencing and assembly methodologies become more pronounced. Moreover, we find generally greater similarity between the human genome assemblies sequenced with capillary-based technologies (GRCh37 and HuRef) than between the human genome assemblies sequenced with massively parallel technologies (NA12878 and YH). Finally, as expected, we find the overall variation in number and content of minimal absent words within a species to be generally smaller than the variation between species.

## Introduction

A minimal absent word of a sequence is a word not found in the sequence; but the removal of its left- or rightmost character uncovers a word that is present in the sequence [1]. Minimal absent words are defined to have at least 3 characters and have been ubiquitously computed in genomes of organisms from all domains of life [2]. The core of a minimal absent word, i.e. the word that remains if its left- and rightmost characters are removed, is a maximal exact repeat. A maximal exact repeat is a perfect repeat, i.e. without gaps or misspellings, that occurs at least twice and which cannot be further extended to either its left- or right-end side without loss of similarity.

For illustration, consider the sequence GCTAACCGATG and its reversed complement CATCGGTTAGC. The set of minimal absent words of these two sequences, concatenated such that artificial words across the boundary between both words are ignored, is 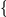 AAA, AAG, AAT, ACA, ACG, ACT, AGA, AGG, AGT, ATA, ATT, CAA, CAC, CAG, CCA, CCC, CCT, CGC, CGT, CTC, CTG, CTT, GAA, GAC, GAG, GCA, GCC, GCG, GGA, GGC, GGG, GTA, GTC, GTG, TAC, TAT, TCA, TCC, TCT, TGA, TGC, TGG, TGT, TTC, TTG, TTT, AGCT, CATG, CCGG, CTAG, GATC, TCGA, TTAA 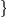, and the set of maximal exact repeats is 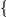 A, C, G, T, AT, CG, GC, TA 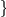.

An important question concerning absent words in genomic sequences is their biological relevance. We have previously investigated the hypothesis of mutational biases (namely, the hypermutability of CpGs) that were proposed to explain the absence in vertebrates [3] of the shortest minimal absent words [4], [5] also explaining the absence of longer minimal absent words. Based on compositional biases, we found no evidence supporting this claim [2]. We have also previously investigated the hypothesis of the inheritance of minimal absent words through a common ancestor in addition to lineage specific inheritance. From the similarity in dinucleotide relative abundances in sets of minimal absent words, we found this claim to be supported only for vertebrates [2]. Moreover, a recent study found an important application for minimal absent words by using them to identify novel splicing events [6].

Having an ever-increasing number of genomes sequenced promotes interest in assessing variation, both within and between species. Here, we assess within species genomic variation in number and content of minimal absent words using four human genome assemblies. We compare two human genome assemblies sequenced with capillary-based technologies, namely, the reference human genome GRCh37 assembly and the HuRef assembly of the genome of Craig Venter, and two human genome assemblies sequenced with massively parallel technologies, namely, the NA12878 assembly from cell line GM12878 and the YH assembly of the genome of a Han Chinese individual. We analyse the distribution of the number of minimal absent words as a function of the minimal absent word length in each human genome assembly; the compositional biases of selected sets of minimal absent words spanning a wide range of word lengths; and the number of common minimal absent words between selected sets of minimal absent words from distinct human genome assemblies. Moreover, as the core of a minimal absent word is a maximal exact repeat, we also analyse the compositional biases at the frontiers of the maximal exact repeats constitutive of minimal absent words, and we attempt an abstract linking between minimal absent words and annotated biological entities by querying a database of consensus sequences of repetitive elements for perfect-alignments to these maximal exact repeats constitutive of minimal absent words.

As minimal absent words are not present in the genome, their use for inferring genomic variation may, at first, appear nonsensical. However, their close association to maximal exact repeats translates into documenting variation in maximal exact repeats and the nucleotides at their frontiers. This close association between minimal absent words and maximal exact repeats is particularly interesting because maximal exact repeats play a key role in massively parallel sequencing, as seeds for the alignment of sequencing reads in genome assembly, and as anchor points in comparisons of closely related genomes [7]; and because repetitive sequences have been experimentally proven to play a prominent role in a highly dynamic structure supporting the uncovered extent of structural variation in the human genome [8].

### Minimal absent words

Let 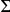 be a finite and ordered set that is called an *alphabet*. Its elements are called *characters* and its cardinality is 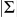. A *string* over the alphabet 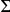 is a finite sequence of elements of 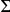. Let 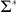 be the set of all strings over 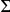, which is equipped with a binary operation obtained by concatenating two sequences. This binary operation is associative. The *empty sequence* 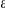 is a neutral element for the operation of concatenation. As a set with a binary operation that is associative and a neutral element is called a *monoid*, the set 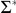 of all strings over the alphabet 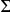 is called the *free monoid* over the set 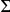. The set of all non-empty words over 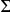, 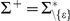, is called the *free semigroup* over 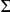.

Let 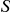 be a string of length 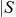 over 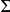 and 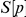 its 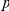 th character, with 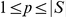. A substring of 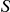 starting at position 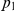 and ending at position 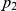 is denoted by 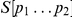, with 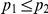. If 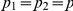, then 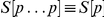. Moreover, 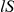 (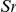) denotes the concatenation of character 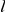 (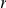) to the left (right) endside of 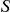, with 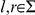. For convenience, consider also two additional characters, 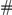 and 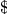, that do not belong to the alphabet 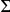. By definition, the character to the left of the first character of string 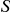 is 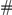, i.e. 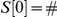, while the character to the right of the last character of string 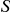 is 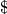, i.e. 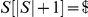.

A maximal repeated pair in 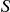 is a pair of identical substrings (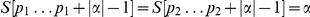) such that the character to the immediate left (right) of one of the substrings is different from the character to the immediate left (right) of the other substring (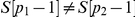 and 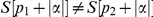). It is represented by a triple (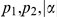), where 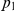 and 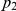 are the starting positions of the two substrings, with 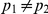. A substring 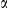 is a *maximal exact repeat* of 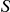 if there is at least a maximal repeated pair in 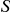 of the form (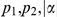) [9].

A string 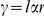 is a *minimal absent word* of 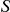 if and only if 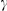 is not a substring of 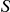, but 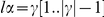 and 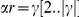 are substrings of 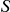. For convenience, we consider 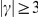. Some theorems concerning minimal absent words have been previously established. **Theorem 1** (proof in [1]): If 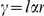 is a minimal absent word of 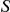, then 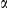 is a maximal exact repeat in 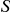. **Theorem 2** (proof in [1]): A string 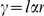 is a minimal absent word of 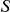 if and only if 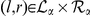 but 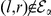, where 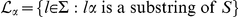, 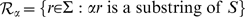 and 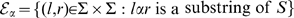. **Theorem 3** (proof in [6]): Any absent word is itself a minimal absent word or a superstring of at least one minimal absent word. **Theorem 4** (proof in [6]): If the reversed complement is also considered for the computation of minimal absent words, then the reversed complement of a minimal absent word is also a minimal absent word.

If 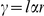 is a minimal absent word of 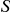, then 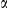 occurs at least twice in 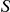 and these occurrences may partially overlap. It is easily verifiable that, as 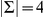 in DNA sequences, the maximum number of minimal absent words associated to a particular maximal exact repeat 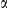 is twelve, and it occurs when 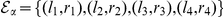, with 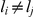 and 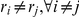. This property implies that frequent maximal exact repeats have a high probability of not generating minimal absent words, because for those frequent maximal exact repeats 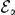 is often equal to 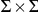.

## Methods

### Four human genome assemblies

We compare four human genome assemblies. The first human genome assembly is the reference GRCh37 assembly build 37.1 from the Genome Reference Consortium, an upgrade on the initial human genome sequenced by the International Consortium using hierarchical shotgun capillary-based methodologies [10]–[12]. The PHRAP and GigAssembler programs were used for assembly. This assembly is organized in chromosomes and is available at the National Center for Biotechnology Information (NCBI) website [13]. The second human genome assembly is the May 2007 HuRef assembly of the genome of J. Craig Venter, sequenced with capillary-based whole-genome shotgun technologies and *de novo* assembled with the Celera Assembler [14]. This assembly is organized in chromosomes and is available at the NCBI website [13]. The third human genome assembly is the NA12878 assembly of DNA from cell line GM12878 [15], sequenced with massively parallel sequencing technologies using Illumina Genome Analyzers and assembled with the ALLPATHS-LG program [15]. The unplaced scaffolds of this assembly are available at the GenBank website [16]. The fourth human genome assembly is the YH assembly of the genome of a Han Chinese, sequenced with massively parallel sequencing technologies using Illumina Genome Analyzers and assembled with the SOAPdenovo assembler [17]. The unplaced scaffolds of this assembly are available at the BGI-Shenzhen website [18].

### Discovering minimal absent words

For discovering minimal absent words, either all chromosomes in a genome are concatenated using a delimiting character that does not belong to the original alphabet to avoid artificial words across the boundaries of the chromosomes (GRCh37 and HuRef assemblies), or all available scaffolds are concatenated using a delimiting character that does not belong to the original alphabet to avoid artificial words across the boundaries of the scaffolds (NA12878 and YH assemblies). The order by which the chromosomes or scaffolds are concatenated is irrelevant (i.e. it does not affect the results). We ignore all sequence ambiguities by replacing every subsequence of ambiguously sequenced nucleotides (i.e. not A, C, G or T) with a delimiting character that does not belong to the original alphabet.

Minimal absent words are found by reading the information in a suffix array. A suffix array is an array of integers 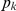, with 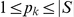 and 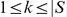, each pointing to the beginning of a suffix of 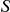, such that 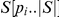 lexicographically precedes 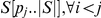. Two auxiliary arrays are used, namely, the longest common prefix (lcp) array, and the left character (bwt) array, the latter corresponding to the Burrows and Wheeler transform [19]. The lcp-array contains the lengths of the longest common prefix between consecutive ordered suffixes, i.e. 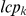 indicates the length of the longest common prefix between 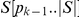 and 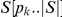, with 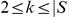. By convention, 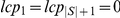. The bwt-array is a permutation of 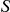 such that 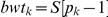 if 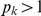, and, by convention, 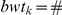 if 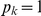, where 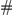 is a character that does not belong to the alphabet 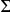. Conceptually, the bwt-array does not provide any additional information, as the left character of any character of 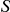 can be determined by direct access to 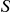. However, the bwt-array allows for sequential memory access, hence improving the performance due to enhanced use of cache [20].

The first part of the algorithm generates all lcp-intervals using the lcp-array and a stack, and is adapted from [21] and [20]. An lcp-interval of lcp-depth 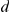 is the interval 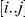, with 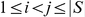, if and only if 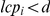; 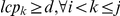; 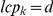, for at least one 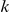 in 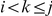; and 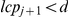. Each lcp-interval delimits a subset of suffixes that start with a common 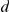-letter prefix 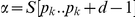, 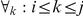. The second part of the algorithm determines if an lcp-interval is left-diverse, i.e. if at least two characters of 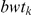 differ, for 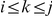. In that case, 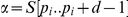 is a maximal exact repeat, as all substrings 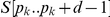 are identical, 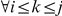. From these maximal exact repeats, all minimal absent words associated to each lcp-interval are computed and then output. See [1] for details on the algorithm.

We define 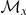 as the set of all minimal absent words 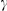 of length 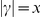. The cardinality of 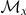 is 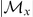. We also define 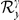 as the set of all unique maximal exact repeats 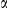 of length 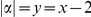 retrieved from set 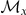 by removing the left- and rightmost characters from each and every minimal absent word in the set. The cardinality of 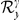 is 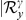.

## Results and Discussion

### Number of minimal absent words

Table 1 displays information on the four human genome assemblies used in this study. We will consider two scenarios: the genome assembly as available and the genome assembly concatenated with its reversed complement. Hence, the noRC data hereafter display results without considering the reversed complement and the withRC data display results considering the reversed complement. The genome size in Table 1 is the number of unambiguous bases, i.e. solely A,C,G or T. The number of minimal absent words (MAWs) indicates their total number in the assembly, i.e. the total for all minimal absent word lengths.

**Table 1:** Four human genome assemblies.

	GRCh37		HuRef		NA12878		YH	
	noRC	withRC	noRC	withRC	noRC	withRC	noRC	withRC
Sequencing	capillary-based		ABI3730xl		Illumina		Illumina	
Assembly	PHRAP & GigAssembler		Celera		ALLPATHS-LG		SOAPdenovo	
Fragment type	chromosomes		chromosomes		scaffolds		scaffolds	
Genome size (bp)	2,861,327,131	5,722,654,262	2,782,339,374	5,564,678,748	2,613,381,835	5,226,763,670	2,218,539,040	4,437,078,080
Number of MAWs	4,217,129,944	8,317,669,642	4,155,779,040	8,235,214,304	3,962,196,417	7,861,209,250	3,546,060,591	7,059,225,195
Longest MAW (bp)	67,633	119,821	9,385	31,117	9,769	34,342	1,281	1,657

GRCh37 is the reference human genome assembly build 37.1, HuRef is the genome of Craig Venter, NA12878 is the human genome assembly from cell line GM12878, and YH is the genome of a Han Chinese individual. Genome size is the number of A,C,G and T base pairs (bp). The number of minimal absent words (MAWs) indicates the total number of minimal absent words in the assembly. The noRC columns display results without considering the reversed complement and the withRC columns display results considering the reversed complement.

**Figure 1:**
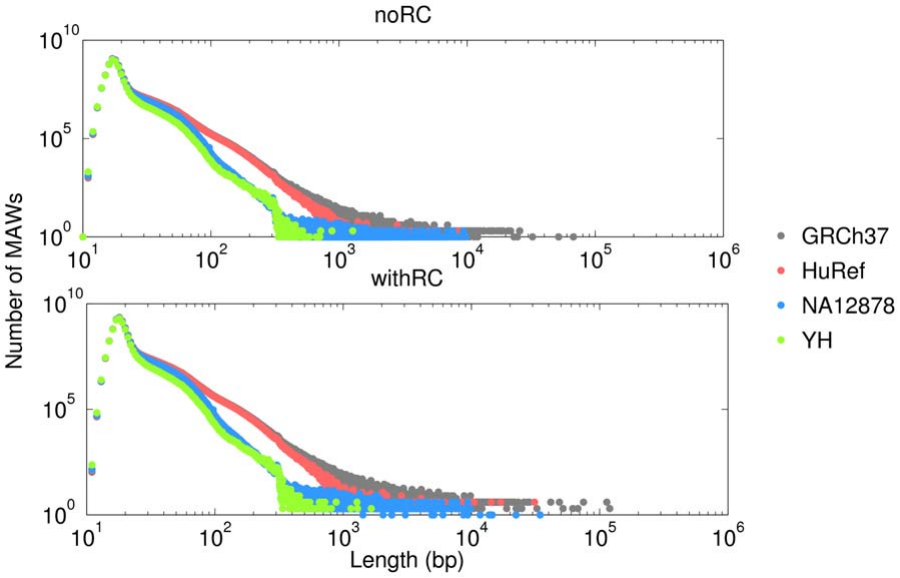
Number of minimal absent words (MAWs) as a function of the minimal absent word length (in units of base pairs) in four human genome assemblies. GRCh37 is the reference human genome assembly build 37.1, HuRef is the genome of Craig Venter, NA12878 is the human genome assembly from cell line GM12878, and YH is the genome of a Han Chinese individual. The upper panel displays results without considering the reversed complement (noRC) and the lower panel displays results considering the reversed complement (withRC).

Figure 1 displays the distribution of minimal absent words in each human genome assembly as a function of the minimal absent word length 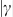. We assess the pairwise distance between distributions of minimal absent words using the total variation distance (TVD), defined as
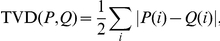
where 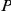 and 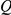 are two probability measures over a finite alphabet, and the term 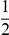 corresponds to the normalization by the two probability distributions [22]. This distance is a L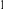-based measure of divergence and it has values in the interval 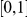, with values closer to the lower limit implying greater similarity, and values closer to the upper implying greater dissimilarity or difference. In order to enhance the differences between these non-stationary distributions, we will consider the distributions divided into four ranges of minimal absent word lengths, namely, 10 bp 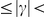 100 bp, 100 bp 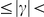 1 kb, 1 kb 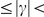 10 kb and 10 kb 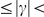 100 kb, where unit bp stands for base pairs and unit kb stands for kilobase pairs. Let all minimal absent words within a given length range and in each human genome assembly be contained in set 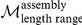, for example, 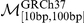. The total variation distance is estimated for each range of minimal absent word lengths and between all pairwise combinations of assemblies. For example, the total variation distance between sets 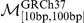 and 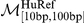 is
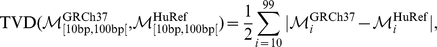
where the sum is over all lengths in the range. Table 1 displays the total variation distance between each pair of distributions for four ranges of minimal absent word lengths. These distributions are most similar for the range of smaller minimal absent words (10 bp 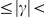 100 bp), as documented by the smaller TVD values, and increasingly more dissimilar for increasingly larger length ranges. The greater similarity between the distributions of minimal absent words in the capillary-based assemblies (GRCh37 and HuRef) in the ranges of 10 bp 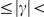 100 bp and 100 bp 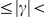 1 kb is clear from both Figure 1 and Table 2. For larger minimal absent words, artefacts from genome sequencing and assembly are likely to dominated over the within species (intra-species) genomic variation. As minimal absent words are constructed over maximal exact repeats, and repetitive sequences are the most difficult to disambiguate, particularly from high-throughput sequencing data, these biases are insurmountable. Moreover, if this total variation distance had not been assessed by range but globally, the more-densely populated regions of the distributions would have overcome the global values of the total variation distance and all detail would have been lost.

**Table 2:** Total variation distance per range of minimal absent word length between the distributions of minimal absent words in four human genome assemblies.

MAW length		noRC			withRC		
		HuRef	NA12878	YH	HuRef	NA12878	YH
10 bp 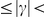 100 bp	GRCh37	0.00320	0.01805	0.05455	0.00220	0.01717	0.05372
	HuRef		0.01530	0.05180		0.01528	0.05183
	NA12878			0.03650			0.03655
100 bp 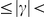 1 kb	GRCh37	0.01953	0.17585	0.08767	0.02160	0.20203	0.11706
	HuRef		0.16258	0.08281		0.18776	0.10833
	NA12878			0.11574			0.10257
1 kb 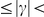 10 kb	GRCh37	0.78940	0.69030	0.99834	0.69294	0.67664	0.99583
	HuRef		0.79879	1		0.74584	1
	NA12878			0.99837			0.99738
10 kb 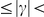 100 kb	GRCh37	–	–	–	1	1	–
	HuRef		–	–		1	–
	NA12878			–			–

The total variation distance is defined as the normalized sum of the absolute differences between the two distributions in each range of minimal absent word (MAW) lengths (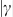). GRCh37 is the reference human genome assembly build 37.1, HuRef is the genome of Craig Venter, NA12878 is the human genome assembly from cell line GM12878, and YH is the genome of a Han Chinese individual. The noRC columns display results without considering the reversed complement and the withRC columns display results considering the reversed complement.

The well-known difficulty in de novo assembly of long and continuous stretches of large and repeat-rich genomes using massively parallel sequencing is here documented by the overall smaller number of discovered minimal absent words in the NA12878 and YH assemblies (Figure 1). Moreover, long repeats are notoriously difficult to assemble and this hinders the number of median-sized, large and very large minimal absent words discovered in genome assemblies using short sequence reads. However, the NA12878 assembly is proof to a successful recent improvement in assembly algorithms for sequencing data from massively parallel platforms [15], here documented by its less scarcity in larger minimal absent words than the YH assembly (Figure 1 and Table 1).

### Content in minimal absent words

We sample the distributions of minimal absent words at specific word lengths, in order to assess the content in minimal absent words of selected sets. We consider minimal absent words of length 11 bp (set 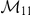), 50 bp (set 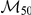), 100 bp (set 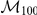), 300 bp (set 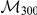) and 1,000 bp (set 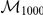). Displayed in Table 3 is the size (cardinality) of each set of minimal absent words, i.e. the total number of minimal absent words in the set, for each human genome assembly.

**Table 3:** Cardinality of selected sets of minimal absent words in four human genome assemblies.

	GRCh37		HuRef		NA12878		YH	
	noRC	withRC	noRC	withRC	noRC	withRC	noRC	withRC
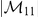	991	106	1,108	128	1,280	142	2,032	234
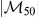	3,249,828	7,311,255	3,116,455	7,066,398	2,114,558	4,928,577	873,006	2,040,419
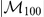	177,208	406,935	166,540	384,855	17,751	50,558	7,217	19,775
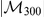	2,027	5,694	1,429	4,056	53	138	66	150
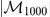	26	62	2	4	3	6	–	–

GRCh37 is the reference human genome assembly build 37.1, HuRef is the genome of Craig Venter, NA12878 is the human genome assembly from cell line GM12878, and YH is the genome of a Han Chinese individual. For each human genome assembly, set 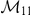 contains all minimal absent words (MAWs) of length 11 bp, set 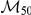 contains all MAWs of length 50 bp, set 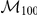 contains all MAWs of length 100 bp, set 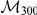 contains all MAWs of length 300 bp, and set 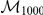 contains all MAWs of length 1,000 bp. The noRC columns display results without considering the reversed complement and the withRC columns display results considering the reversed complement.

**Figure 2:**
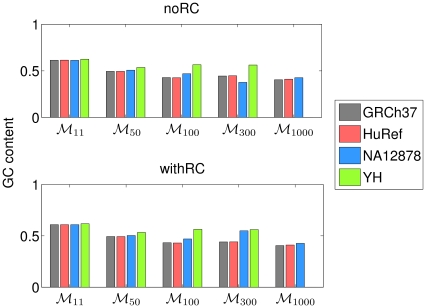
GC content of selected sets of minimal absent words in four human genome assemblies. The GC content is the overall fraction of G plus C nucleotides in each set. GRCh37 is the reference human genome assembly build 37.1, HuRef is the genome of Craig Venter, NA12878 is the human genome assembly from cell line GM12878, and YH is the genome of a Han Chinese individual. For each human genome assembly, set 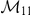 contains all minimal absent words (MAWs) of length 11 bp, set 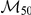 contains all MAWs of length 50 bp, set 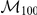 contains all MAWs of length 100 bp, set 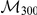 contains all MAWs of length 300 bp, and set 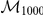 contains all MAWs of length 1,000 bp. The upper panel displays results without considering the reversed complement (noRC) and the lower panel displays results considering the reversed complement (withRC).

The first parameter of variation in content of minimal absent words is the compositional bias (GC content) of the selected sets of minimal absent words in each human genome assembly, displayed in Figure 2. The GC content is the overall fraction of G plus C nucleotides in each set. As before [2], these compositional biases are not uniform throughout the different sets of minimal absent words, though, as expected, this intra-species (within species) variation is generally smaller than its inter-species (between species) counterpart [2]. For example, consider sets 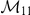 in the scenario with the reversed complement. The GC content of these sets of minimal absent words is 0.6090 for the GRCh37 assembly, 0.6080 for the HuRef assembly, 0.6082 for the NA12878 assembly, and 0.6177 for the YH assembly. However, previously reported GC content values for sets 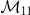 of some eukaryotes [2] are 0.6456 for the budding yeast *Saccharomyces cerevisiae* strain S228C (SGD release 1, [23]), 0.7970 for the thale cress *Arabidopsis thaliana* (AGI release 7.2, [24]), 0.7038 for the worm *Caenorhabditis elegans* (WormBase release 170, [25]), 0.6923 for the fruit fly *Drosophila melanogaster* (FlyBase release 5, [26]), 0.6070 for the chicken *Gallus gallus* (build 2.1, [13]), 0.6172 for the mouse *Mus musculus* (build 37.1, [13]), and 0.6176 for the chimpanzee *Pan troglodytes* (build 2.1, [13]). Hence, the module of the difference in GC content between the human genome assemblies is generally smaller than the difference between a human genome assembly and other species. As the overall GC content is a coarse measure of similarity (conversely, variability), the difference between human genome assemblies is not always smaller than that between human genome assemblies and other vertebrates (e.g. the GRCh37 and YH assemblies versus the GRCh37 assembly and the chimpanzee). However, this difference becomes more pronounced for organisms evolutionary more distant (e.g. the fruit fly, worm, or the budding yeast).

**Figure 3:**
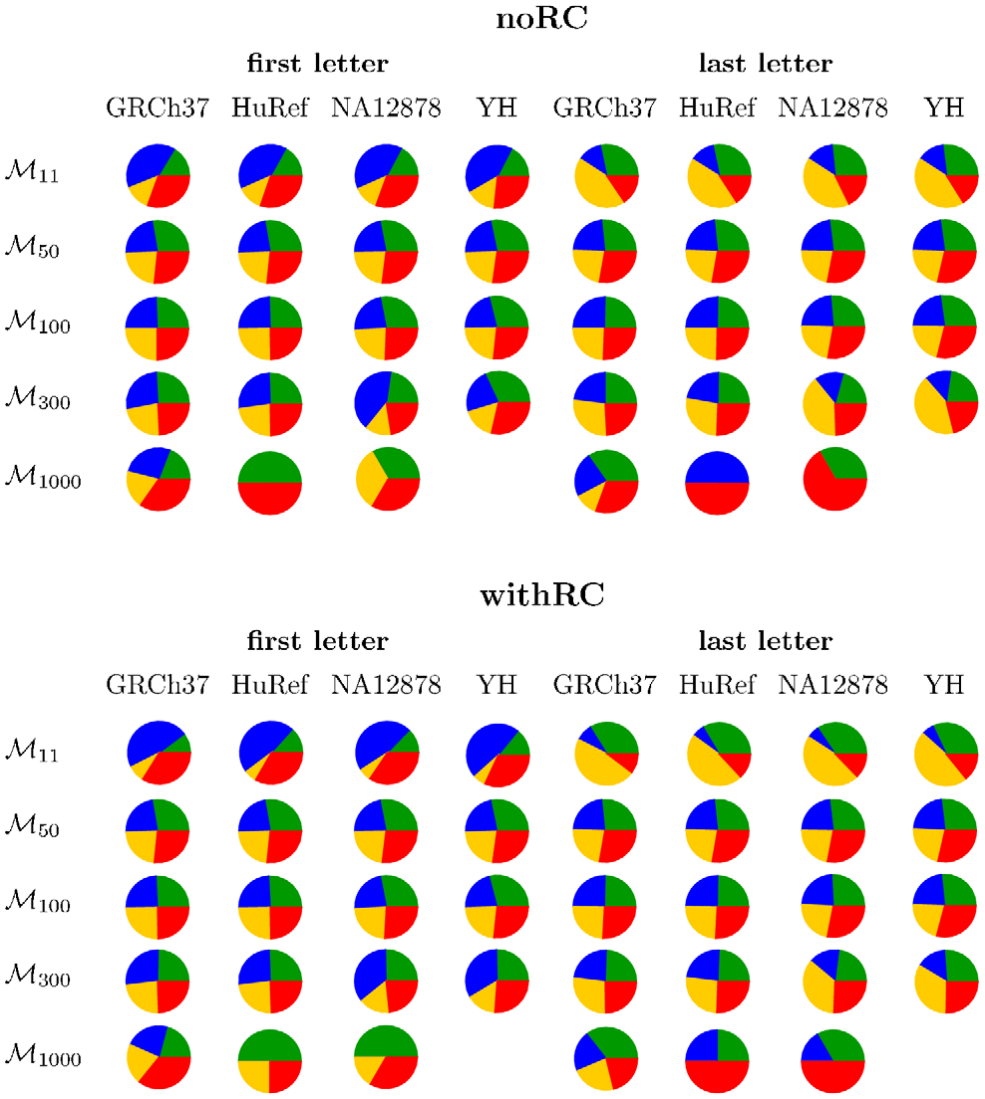
Compositional nucleotide biases in the first and last letters of the minimal absent words in selected sets of minimal absent words in four human genome assemblies. Green slices represent the fraction of A nucleotides, blue slices represent the fraction of C nucleotides, yellow slices represent the fraction of G nucleotides, and red slices represent the fraction of T nucleotides. GRCh37 is the reference human genome assembly build 37.1, HuRef is the genome of Craig Venter, NA12878 is the human genome assembly from cell line GM12878, and YH is the genome of a Han Chinese individual. For each human genome assembly, set 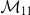 contains all minimal absent words (MAWs) of length 11 bp, set 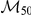 contains all MAWs of length 50 bp, set 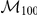 contains all MAWs of length 100 bp, set 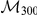 contains all MAWs of length 300 bp, and set 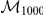 contains all MAWs of length 1,000 bp. The noRC area displays results without considering the reversed complement and the withRC area displays results considering the reversed complement.

As variation in minimal absent words represents variation in maximal exact repeats and the nucleotides at their frontiers, Figure 3 displays the nucleotide compositional biases of the first and last letters of the minimal absent words in selected sets. Again, these compositional biases are more dissimilar in sets of minimal absent words of larger word length.

**Figure 4:**
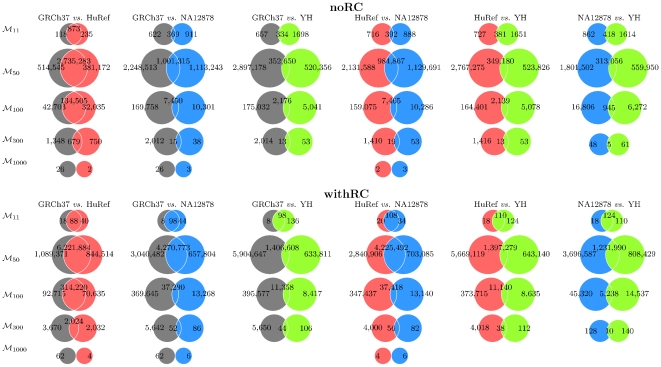
Number of minimal absent words at the intersection of selected sets of minimal absent words in four human genome assemblies. GRCh37 is the reference human genome assembly build 37.1 (grey circles), HuRef is the genome of Craig Venter (pink circles), NA12878 is the human genome assembly from cell line GM12878 (blue circles), and YH is the genome of a Han Chinese individual (green circles). For each human genome assembly, set 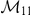 contains all minimal absent words (MAWs) of length 11 bp, set 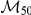 contains all MAWs of length 50 bp, set 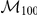 contains all MAWs of length 100 bp, set 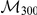 contains all MAWs of length 300 bp, and set 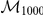 contains all MAWs of length 1,000 bp. The noRC area displays results without considering the reversed complement and the withRC area displays results considering the reversed complement.

The second and foremost parameter of variation in content of minimal absent words between human genome assemblies is the number of common minimal absent words between two sets of minimal absent words, displayed at the intersection of both sets in the Venn diagrams of Figure 4. This set content similarity is further summarized by the Jaccard similarity indexes displayed in Table 4. The Jaccard similarity index is the ratio between the intersection and the union of two sets, hence its possible values are between 0 and 1, with the latter resuming greater similarity [27]. As with the number of minimal absent words, the comparison of the content of selected sets of minimal absent words renders increasing dissimilarity as the length of the minimal absent word increases. Moreover, the two human genome assemblies more similar overall in minimal absent word content are the GRCh37 and HuRef assemblies, whereas the overall similarity for the remaining pairwise comparisons is markedly inferior. Again, the intra-species variation with respect to this parameter is smaller than its inter-species counterpart. Considering sets 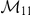 in the scenario with the reversed complement, the Jaccard similarity index between the GRCh37 human genome assembly and three vertebrates is 0.015 for the chicken *Gallus gallus* (build 2.1, [13]), 0.014 for the mouse *Mus musculus* (build 37.1, [13]), and 0.181 for the chimpanzee *Pan troglodytes* (build 2.1, [13]). These values are clearly smaller than those reported in Table 4 for sets 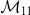 (withRC columns) between any pair of human genome assemblies.

**Table 4:** Jaccard similarity index for pairwise comparisons of selected sets of minimal absent words in four human genome assemblies.

	GRCh37		GRCh37		GRCh37		HuRef		HuRef		NA12878	
	*vs.*		*vs.*		*vs.*		*vs.*		*vs.*		*vs.*	
	HuRef		NA12878		YH		NA12878		YH		YH	
	noRC	withRC	noRC	withRC	noRC	withRC	noRC	withRC	noRC	withRC	noRC	withRC
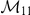	0.712	0.603	0.194	0.653	0.124	0.405	0.196	0.667	0.138	0.437	0.144	0.492
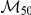	0.753	0.763	0.229	0.536	0.094	0.177	0.232	0.544	0.096	0.181	0.117	0.215
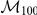	0.643	0.658	0.040	0.089	0.012	0.027	0.042	0.094	0.012	0.028	0.039	0.080
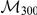	0.245	0.262	0.007	0.009	0.006	0.008	0.013	0.014	0.009	0.009	0.044	0.036
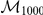	0	0	0	0	–	–	0	0	–	–	–	–

The Jaccard similarity index is the ratio between the intersection and the union of the two sets. GRCh37 is the reference human genome assembly build 37.1, HuRef is the genome of Craig Venter, NA12878 is the human genome assembly from cell line GM12878, and YH is the genome of a Han Chinese individual. For each human genome assembly, set 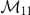 contains all minimal absent words (MAWs) of length 11 bp, set 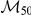 contains all MAWs of length 50 bp, set 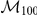 contains all MAWs of length 100 bp, set 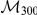 contains all MAWs of length 300 bp, and set 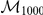 contains all MAWs of length 1,000 bp. The noRC columns display results without considering the reversed complement and the withRC columns display results considering the reversed complement.

### Maximal exact repeats constitutive of minimal absent words

Finally, we attempt an abstract linking between minimal absent words and annotated biological entities by querying a database of consensus sequences of repetitive elements for perfect-alignments to these maximal exact repeats constitutive of minimal absent words. Displayed in Table 5 is the size (cardinality) of each set of unique maximal exact repeats obtained from the respective sets of minimal absent words. For example, set 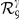 contains all unique maximal exact repeats of length 9 bp obtained by removing the left- and rightmost characters of each and every minimal absent word of length 11 bp in set 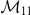. These 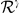 sets, which contain solely one copy of the maximal exact repeats constitutive of minimal absent words, may be smaller than their respective counterparts containing all maximal repeats of a given repeat length.

**Table 5:** Cardinality of sets of maximal exact repeats obtained from selected sets of minimal absent words in four human genome assemblies and number of perfect-alignment matches to repeats in Repbase.

	GRCh37		HuRef		NA12878		YH	
	noRC	withRC	noRC	withRC	noRC	withRC	noRC	withRC
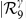	932	104	1,044	125	1,194	139	1,878	229
Total	465	47	520	68	689	59	1,052	101
Unique	244	43	257	56	292	52	384	83
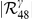	2,564,066	5,746,703	2,459,228	5,555,328	1,719,083	3,968,192	715,704	1,652,986
Total	81,530	108,400	80,796	107,694	59,655	86,566	25,029	34,576
Unique	403	485	394	485	388	478	292	384
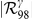	133,964	306,954	125,245	288,243	16,057	44,828	6,342	17,143
Total	16,785	24,229	16,338	23,725	3,454	6,526	1,883	3,448
Unique	71	97	70	97	75	94	39	44
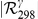	1,891	5,198	1,327	3,655	45	118	61	128
Total	181	568	148	471	1	5	0	0
Unique	3	5	3	4	1	2	0	0
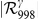	26	62	1	3	3	6	–	–
Total	1	1	0	0	0	0	–	–
Unique	1	1	0	0	0	0	–	–

GRCh37 is the reference human genome assembly build 37.1, HuRef is the genome of Craig Venter, NA12878 is the human genome assembly from cell line GM12878, and YH is the genome of a Han Chinese individual. For each human genome assembly, set 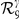 contains all unique maximal exact repeats (MERs) of length 9 bp obtained from the minimal absent words (MAWs) in set 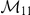, set 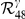 contains all unique MERs of length 48 bp obtained from the MAWs in set 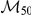, set 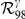 contains all unique MERs of length 98 bp obtained from the MAWs in set 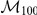, set 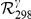 contains all unique MERs of length 298 bp obtained from the MAWs in set 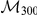, and set 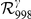 contains all unique MERs of length 998 bp obtained from the MAWs in set 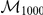. Total values include all unique perfect-alignment matches times their multiplicity. The noRC columns display results without considering the reversed complement and the withRC columns display results considering the reversed complement.

We survey the maximal exact repeats constitutive of minimal absent words for similarity to repeats in Repbase [28], a comprehensive database of consensus sequences of repetitive elements, for perfect-alignment matches. A total of 1,168 repeats for the human genome and respective evolutionary ancestry were retrieved in FASTA format from this database. The matches reported are exact, i.e. there is a perfect-alignment between the maximal exact repeat and the repeat in the database, though possibly partial, i.e. the repeat in the database may be larger than the maximal exact repeat. Also, only one match per pair of maximal exact repeat/repeat in database is reported. Also displayed in Table 5 is the total number of matches for each set of maximal exact repeats (total), then filtered to discount the multiplicity of each match (unique). The ratio of the total number of matches to the cardinality of the 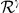 set provides an estimate of the large number of maximal exact repeats at the core of minimal absent words that do not match any annotated repeat in Repbase. Moreover, the ratio of the unique matches to the size of the database (1,168 repeats) provides a complementary estimate of this pool of unannotated repetitive sequences. As with other parameters of variation assessed before, there is a dependency of the percentage of perfect-alignment matches with the length of the minimal absent words (hence, of the maximal exact repeats) and with the human genome assembly, the latter varying overall less than the former.

**Figure 5:**
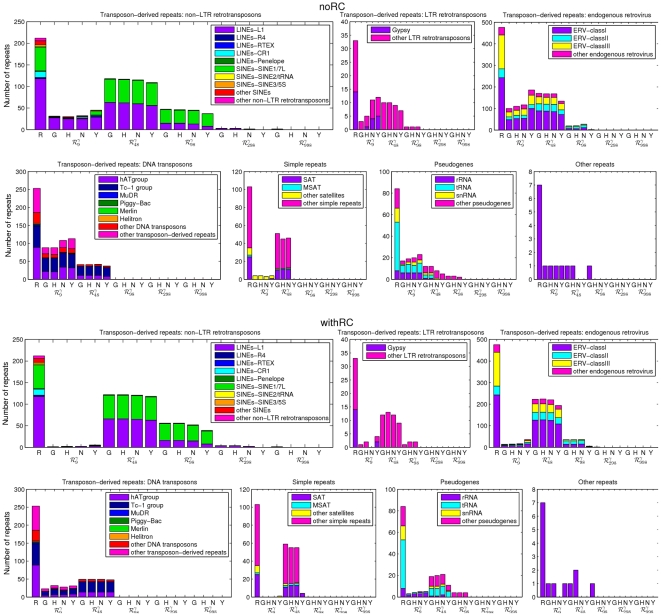
Repeat-class-discriminated number of perfect-alignment matches of maximal exact repeats constitutive of selected sets of minimal absent words in four human genome assemblies to repeats in Repbase. Each repeat class is identified by the title of the respective subplot and subdivided into repeat families by a color scheme. R bars represent the number of repeats in the family annotated in Repbase. G bars represent the number of perfect-alignment matches of the MERs in set 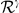 from the GRCh37 assembly to the repeats in Repbase, H bars represent the corresponding matches for the HuRef assembly, N bars represent the corresponding matches for the NA12878 assembly, and Y bars represent the corresponding matches for the YH assembly. GRCh37 is the reference human genome assembly build 37.1, HuRef is the genome of Craig Venter, NA12878 is the human genome assembly from cell line GM12878, and YH is the genome of a Han Chinese individual. For each human genome assembly, set 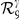 contains all unique maximal exact repeats (MERs) of length 9 bp obtained from the minimal absent words (MAWs) in set 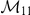, set 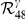 contains all unique MERs of length 48 bp obtained from the MAWs in set 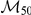, set 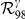 contains all unique MERs of length 98 bp obtained from the MAWs in set 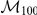, set 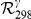 contains all unique MERs of length 298 bp obtained from the MAWs in set 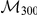, and set 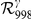 contains all unique MERs of length 998 bp obtained from the MAWs in set 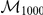. The upper panels (noRC) display results without considering the reversed complement and the lower panels (withRC) display results considering the reversed complement.

To make evident which repeat classes and families are associated to these matches, Figure 5 displays the repeat-class-discriminated numbers for each human genome assembly, with the repeat class identified by the title of the respective subplot, and complemented by a color scheme to discriminate the repeat families in the class. The five major classes of repetitive sequences in the human genome are transposon-derived (or interspersed) repeats, processed pseudogenes, simple sequence repeats, segmental duplications, and tandem repeats [10], but we do not address segmental duplications here. In mammals, almost all transposon-derived repeats can be classified into four classes, namely, long interspersed elements (LINEs), short interspersed elements (SINEs), LTR retrotransposons, and DNA transposons. LINEs are autonomous transposons of about 6 kb long and SINEs are short nonautonomous transposons of about 100–400 bp long. LINE and SINE lineages have extremely long lives, the former, with only one family still active (LINE1), being the most ancient and typically present in AT-rich areas of the genome; whereas the latter, with only one family still active (Alus), typically exists in GC-rich areas of the genome (though recent Alus show a preference for AT-rich areas, whereas progressively older Alus show a progressively stronger bias towards GC-rich areas). Although a variety of LTR retrotransposons exist, only the vertebrate-specific endogenous retroviruses (ERVs) appear to have been active in the human genome. Mammalian retroviruses fall into three classes (I–III), each comprising many families with independent origins. DNA transposons, which resemble bacterial transposons, can be subdivided into many families with independent origins and tend to have short life spans within a species. LTR transposons and DNA transposons show a more uniform distribution along the human genome, with respect to GC content, except for the most GC-rich regions, where their presence is minor. Moreover, DNA transposon copies in AT-rich areas tend to be younger than those in more GC-rich areas [10].

The data in Figure 5 makes evident the sequence similarity of the maximal exact repeats constitutive of minimal absent words to distinct repeat classes, hence to distinct functional and evolutionary roles. These preferences can be partially explained, on the one hand, by the constraints imposed by the length of the maximal exact repeat (e.g. if SINEs are typically 100–300 bp long, it is not expected that maximal repeats in set 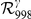 will match any repeats in that class), and, on the other hand, by the compositional biases of the maximal exact repeats (e.g. due to the high GC content of set 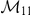, the DNA transposons matched are expected to be older than those of sets with lower GC content). Again, this variation in repeat classes is more pronounced between different sets of minimal absent words (hence, of maximal exact repeats) than between human genome assemblies.

This query of Repbase for perfect-alignments to the maximal exact repeats constitutive of minimal absent words does not render the attempted abstract linking an effective identity, as the position of the maximal exact repeats would have to match that of the repeats in the database and this was not here investigated.

### Conclusions

Minimal absent words have been computed in genomes of organisms from all domains of life. While the inter-species variation in number and content of minimal absent words had been previously addressed, here we explore intra-species variation using four human genome assemblies, thus contributing to the catalogue of human genomic variation. We compare two human genome assemblies sequenced with capillary-based technologies, namely, the reference human genome GRCh37 assembly and the HuRef assembly of the genome of Craig Venter, and two human genome assemblies sequenced with massively parallel technologies, namely, the NA12878 assembly from cell line GM12878 and the YH assembly of the genome of a Han Chinese individual. Without the constraints imposed by the smaller prokaryotic genomes, here we investigate sets of minimal absent words spanning a wide range of word lengths. We analyse the distribution of the number of minimal absent words as a function of the minimal absent word length in each human genome assembly; the compositional biases of selected sets of minimal absent words spanning a wide range of word lengths; and the number of common minimal absent words between selected sets of minimal absent words from distinct human genome assemblies. We find that, as expected, the overall intra-species (within species) variation in number and content of minimal absent words is generally less pronounced than their inter-species (between species) counterpart. Moreover, we find the variation in number and content of minimal absent words between human genome assemblies more significant for large and very large minimal absent words, where the biases of sequencing and assembly methodologies for large and repeat-rich genomes become more evident. As minimal absent words are constructed over maximal exact repeats, and repetitive sequences are the most difficult to disambiguate, particularly from high-throughput sequencing data, these biases are insurmountable. Finally, we find generally greater similarity between the human genome assemblies sequenced with capillary-based technologies (GRCh37 and HuRef) than between the human genome assemblies sequenced with massively parallel technologies (NA12878 and YH).

As the core of a minimal absent word is a maximal exact repeat, we also analyse the compositional biases at the frontier of the maximal exact repeats constitutive of minimal absent words, and we attempt an abstract linking between minimal absent words and annotated biological entities by querying a database of consensus sequences of repetitive elements for perfect-alignments to the maximal exact repeats constitutive of minimal absent words. Due to their relevance in massively parallel sequencing and comparative genomics, it is important to distinguish maximal exact repeats that are homologous from those whose similarity is spurious, i.e. occurs by chance alone. We believe the combinatorial scheme over single-nucleotide mismatches at the frontiers of maximal exact repeats that defines minimal absent words may render minimal absent words an interesting fingerprint of maximal exact repeat homology, to be investigated in future studies.
